# Antifungal susceptibility and molecular characterization of clinical and environmental isolates of *Schizophyllum commune*

**DOI:** 10.1128/jcm.01442-25

**Published:** 2026-02-18

**Authors:** Grégoire Pasquier, Pierre-Olivier Harmand, Laura Le Feur, Emilie Guemas, Anne Pauline Bellanger, Danièle Maubon, Anne Favel, Claire Cottrel, Lilia Hasseine, Marcela Sabou, Eric Dannaoui, Arnaud Fekkar, Anne-Cécile Normand, Jean-Pierre Gangneux, Laurence Delhaes, Milène Sasso, Laurence Lachaud

**Affiliations:** 1 Laboratoire de Parasitologie-Mycologie, CHU de Montpellier 26905 , Montpellier, France; 2 UMR MIVEGEC, Université de Montpellier 27037, Montpellier, France; 3 Service de Parasitologie-Mycologie, Hôpital Purpan, Centre Hospitalier Universitaire de Toulouse 36760, Toulouse, France; 4 Parasitology-Mycology Department, Besancon University Hospital 55049 , Besancon, France; 5 Translational Innovation in Medicine and Complexity, Centre National de la Recherche Scientifique, Université Grenoble Alpes, Domaine de la Merci, Centre Hospitalier Universitaire Grenoble Alpes, Service de Parasitologie-Mycologie 442670 , La Tronche, France; 6 INRAE, Aix Marseille Univ, CIRM-CF, Centre International des Ressources Microbiennes-Champignons Filamenteux, Marseille, France; 7 Microbiology Laboratory, Université de Lorraine, CHRU-Nancy, Nancy, France; 8 Laboratoire de Parasitologie-Mycologie, CHU de Nice, Nice, France; 9 Strasbourg Institute of Parasitology and Tropical Pathology, Strasbourg University Hospital, Strasbourg, France; 10 Unité de Parasitologie-Mycologie, Service de Microbiologie, Hôpital Necker, AP-HP, Paris, France; 11 Department of Parasitology and Mycology, Assistance Publique-Hôpitaux de Paris (AP-HP), La Pitié-Salpêtrière Hospital, Paris, France; 12 Laboratoire de Parasitologie-Mycologie, CNR des aspergilloses chroniques-Nord, CHU de Rennes, European Excellence Center in Medical Mycology – ECMM EC 36684, Rennes, France; 13 Laboratoire de Parasitologie-Mycologie, CNR des aspergilloses chroniques, CHU de Bordeaux 36836, Bordeaux, France; 14 Laboratoire de Parasitologie-Mycologie, CHU de Nîmes 36672, Nîmes, France; University of Utah, Salt Lake City, Utah, USA

**Keywords:** *Schizophyllum commune* or *radiatum*, antifungal susceptibility, standardization, EUCAST, CLSI

## Abstract

*Schizophyllum commune* is a cosmopolitan, saprophytic basidiomycete known to cause respiratory tract infections following spore inhalation. Management of such infections remained poorly defined, and antifungal susceptibility testing may provide valuable guidance for therapeutic decisions. However, existing data are limited, and protocols are not optimized for non-sporulating molds such as *S. commune*, requiring methodological adaptations. This study aimed to (i) perform molecular characterization of isolates and (ii) determine antifungal susceptibility profiles of a collection of *S. commune isolates* using both Clinical and Laboratory Standards Institute (CLSI) and European Committee on Antimicrobial Susceptibility Testing (EUCAST) reference methods. A total of 113 fungal isolates were included, comprising environmental (*n* = 31), clinical French isolates (*n* = 74), and strains from fungal international collections (*n* = 8). Species identification was confirmed via the large subunit (LSU) region of the ribosomal DNA sequencing and by matrix-assisted laser desorption/ionization time-of-flight mass spectrometry. Phylogenetic analysis was conducted on a subset of 20 isolates using partial sequences of three genes: LSU, EF-1α, and RPB2. Modifications to the CLSI and EUCAST broth microdilution methods included: inoculum preparation using standardized culture slices fragmented by bead-beating, incubation at 35°C for 96 h and endpoints reading at 100% of inhibition. Phylogenetic analysis confirmed that all tested isolates were *S. commune* and not *S. radiatum*. Amphotericin B (AMB) and voriconazole (VOR) demonstrated low geometrical mean minimal inhibitory concentrations (MICs) across both methods (EUCAST: AMB = 0.39 µg/mL, VOR = 0.24 µg/mL; CLSI: AMB = 0.1 µg/mL, VOR = 0.2 µg/mL). In contrast, terbinafine exhibited high MIC values (>8 µg/mL) in both protocols. Notable discrepancies were observed for posaconazole (POS), isavuconazole (ISA), and itraconazole (ITR), with MICs approximately two dilution steps higher in EUCAST compared to CLSI (POS: 4.22 µg/mL vs 0.9 µg/mL, ISA: 3.64 µg/mL vs 0.74 µg/mL, and ITR: 3.9 µg/mL vs 0.81 µg/mL, respectively). After standardization of both methods for this non-sporulating mold, VOR and AMB have the lowest MIC values.

**IMPORTANCE:**

*Schizophyllum commune* is a fungal pathogen increasingly associated with respiratory infections, yet therapeutic guidance remains unclear. This study provides the largest collection to date of clinical and environmental isolates (113 total) and applies standardized antifungal susceptibility testing using adapted EUCAST and CLSI protocols for this non-sporulating species. The results show that amphotericin B and voriconazole are the most active agents *in vitro*, while terbinafine is ineffective. These findings are critical for informing treatment decisions and interpreting susceptibility tests, especially in the absence of established guidelines. By introducing a reproducible methodology and delivering clinically relevant data, this work addresses a gap in medical mycology and supports improved management of rare fungal infections.

## INTRODUCTION

*Schizophyllum commune* Fr. (Basidiomycota, Schyzophyllaceae) is a saprophytic, cosmopolitan split-gill mushroom capable of causing respiratory tract infections following inhalation of its spores. The most frequently reported clinical manifestations are sinusitis and allergic bronchopulmonary mycosis (ABPM) (1). In 2013, 71 cases were reviewed: 45 (63%) were bronchopulmonary, 22 (31%) were sinusitis, and 4 were extrapulmonary (1). It should be noted that two rhinosinusitis cases were reported in COVID-19 patients (2, 3). Although human infections are relatively rare, *S. commune* is the most frequently reported basidiomycete in clinical settings, accounting for approximately half of all documented cases (4). Diagnosis is often challenging as it is a non-sporulating mold forming non-specific white cottony colonies in culture (5) which may easily be overlooked by medical mycologists. Historically, definite identification relied on the observation of basidiospore production in rare dikaryotic strains (6). However, most of the clinical strains are monocaryotic, and current identification methods primarily include molecular sequencing (5) and matrix-assisted laser desorption/ionization time-of-flight (MALDI-TOF) mass spectrometry (7). Therapeutic management of *S. commune* infections remains poorly defined and is largely based on individual case reports and small case series. The joint guidelines issued by the European Confederation of Medical Mycology (ECMM), the International Society for Human and Animal Mycology (ISHAM), and the American Society for Microbiology (ASM) for rare molds (8) recommend amphotericin B (AMB) as first-line therapy, with a step-down to oral posaconazole (POS) or voriconazole (VOR) as an alternative option for invasive disease. Sinusitis is typically managed surgically, with or without adjunctive antifungal therapy (9) and ABPM is generally treated with a combination of corticosteroids and itraconazole (ITR) (10).

Given the increasing number of reported *S. commune* infections (4) and the lack of standardized treatment protocols, antifungal susceptibility testing may provide valuable guidance for therapeutic decision-making, as recommended by ECMM-ISHAM and ASM guidelines (8). However, interpretation of minimum inhibitory concentrations (MICs) should be approached with caution due to the absence of established breakpoints and epidemiological cut-off values. Available susceptibility data are limited and primarily derived from individual case reports (11–19). Only three studies have evaluated five or more isolates (up to 30) using modified Clinical and Laboratory Standards Institute (CLSI) methods (20–22). These studies consistently reported low MICs for AMB and high MICs for fluconazole and 5-fluorocytosine. MICs for ITR, VOR, and POS were generally low, with the exception of one study (22) reporting *S. radiatum* isolates exhibiting higher MICs. *S. radiatum* Fr. is a sibling species of *S. commune*, which was differentiated based on subtle micromorphological traits, including the size of basidiospores and of the abhymenial hairs (23, 24), although these distinctions are not universally accepted (25). Ribosomal DNA (rDNA) sequencing (26) does not allow them to be reliably distinguished. However, phylogenetic analysis based on the partial sequencing of three genes has demonstrated a good discriminatory power (22).

To ensure the reliability of antifungal susceptibility results for these species, testing methods must be adapted, standardized, and validated using 113 isolates. The objectives of the present multicenter study were as follows: (i) to perform molecular characterization on a subset of isolates to confirm their identification as *S. commune* or *S. radiatum* and (ii) to assess, following standardization, the antifungal susceptibility of 113 clinical or environmental isolates to six antifungal agents using both the European Committee on Antimicrobial Susceptibility Testing (EUCAST) and CLSI reference microdilution methods.

## MATERIALS AND METHODS

### Fungal isolate collection and identification

**Fig 1:**
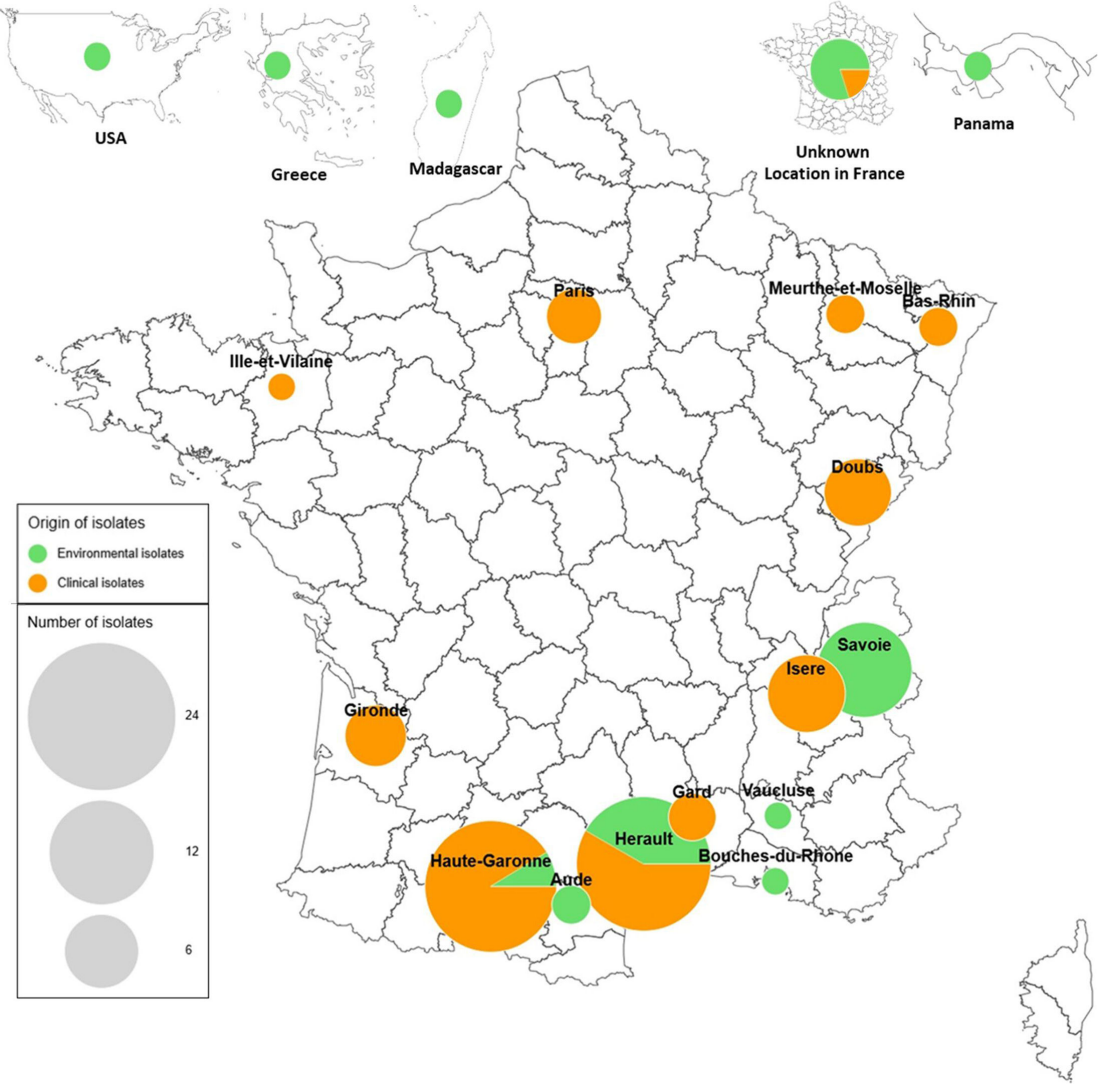
National and regional origins of the 113 isolates included in the study. The map was designed with ArctiqueR online software version 8.0.12267.

Fungal isolates (*n* = 113) were obtained through three distinct sources (Fig. 1). (i) Environmental samples (*n* = 31) of split-gilled mushrooms were collected by the authors or collaborators from decaying wood in various locations across southern France, except for one sample from Greece. (ii) Clinical isolates (*n* = 74) were recovered from 10 French mycology departments of university hospitals. They had been isolated from various respiratory tract samples, mainly sinus samples (52.7%, 39/74), expectoration (12.2%, 9/74), tracheal or bronchial aspirates (10.8%, 8/74), and broncho-alveolar lavage fluid (9.5%, 7/74). Ten samples were of undetermined origin (13.5%). (iii) Reference strains (*n* = 8) were acquired from international fungal culture collections: Center International de Ressources Microbiennes – Champignons Filamenteux (CIRM-CF) of France and the Westerdijk Fungal Biodiversity Institute (WI-KNAW) culture collection of the Netherlands.

Species-level identification of *S. commune* was confirmed using two complementary approaches: MALDI-TOF mass spectrometry (Brucker) with the MSI-II database (27) and molecular identification via the large subunit (LSU) of the rDNA sequencing (28).

### MALDI-TOF identification

All 113 isolates were identified as *S. commune* by MALDI-ToF using the MSI-2 database with a confidence score ≥20. The only exception was the CBS 301.32 *S*. *radiatum* strain. In this last case, the identification score (19.09) did not reach the required minimum of 20. Currently, the MSI-2 database includes 17,563 spectra from 1,702 fungal species, including 20 spectra of *S. commune*, but none of *S. radiatum*.

### Molecular characterization

Genomic DNA was extracted using the QIAamp DNA mini kit (QIAGEN), following the manufacturer’s protocol, with an initial mechanical disruption step using bead-beating (MagNA Lyser Roche). All 113 isolates underwent molecular identification via the LSU region sequencing using primers NL-1 and NL-4 (28). For phylogenetic analysis, two additional partial genes—translation elongating factor 1α (EF-1α) and RNA polymerase II second-largest subunit (RPB2) (22)—were amplified in a subset of 20 isolates selected to represent the geographical and clinical/environmental diversity of the panel. The primers were specifically designed for *S. commune* and their sequences are presented in Table S1.

Sequencing was performed using ABI3500 Genetic Analyzers and the BigDye Terminator v3.1 Cycle Sequencing. Consensus sequences were assembled from forward and reverse sequences using BioEdit 7.7. All the sequences were deposited in GenBank (Table S2).

### Phylogenetic tree

Phylogenetic relationships were inferred following the methodology described by Siqueira et al. (22). Briefly, concatenated sequences of three genetic markers—LSU, EF-1α, and RPB2—were obtained from three sources: (i) 18 isolates obtained in the present study, (ii) two reference strains (CBS301.32 and CBS476.64), and (iii) a set of previously sequenced isolates (20).

Multiple sequence alignments were performed using the MUSCLE algorithm in Mega11 software (29). The best-fit nucleotide substitution model was determined using both MEGA11 and jModelTest v2.1.10, with both analyses selecting the Tamura-Nei model with a discrete gamma distribution and a proportion of invariant sites (TN93+I+G or TrN+I+G).

Maximum likelihood (ML) analysis was conducted in Mega11, with branch support assessed via 500 bootstrap replicates; values ≥70 were considered significant. Bayesian inference was performed using MrBayes 3.2.7a, with Markov chain Monte Carlo simulations run for 3,000,000 generations, sampling every 100 generations and diagnostics every 1,000. Posterior probabilities ≥0.95 were considered statistically significant.

### Antifungal susceptibility testing

MICs for six antifungal agents were determined for all 113 isolates using both adapted EUCAST and CLSI reference microdilution methods. The antifungal agents from Sigma-Aldrich were tested: AMB, VOR, POS, ITR, isavuconazole (ISA), and terbinafine (TER).

Due to the non-sporulating nature of *S. commune*, modifications to standard EUCAST (document 9.4) (30) and CLSI (document M38) (31) protocols for filamentous fungi were required. A comparative evaluation of inoculum preparation methods was conducted using seven isolates and three approaches. (i) Cover the cultures with 10 ml of sterile water, then transfer them to a sterile tube as described in the EUCAST 9.4 document (30). (ii) Collect one cm^2^ of fresh culture in 1.2 mL sterile water. Then, vortex manually for 15 seconds at ~2,000 rpm. Finally, perform a 1:10 dilution for EUCAST and a 1:50 dilution for CLSI. (iii) Use the same method as method ii, except perform the vortex step using a MagNA Lyser centrifuge for 60 s at 2,000 rpm using ceramic beads (MagNA Lyser Green Beads, Roche). The third method was selected for standardization testing. Repeatability (duplicate testing on the same day, *n* = 24–30 strains) and reproducibility tests (triplicate testing on distinct days, *n* = 8 strains) were assessed for all six antifungal agents.

All isolates were cultured for 7–10 days at 30°C on benomyl supplemented Sabouraud dextrose agar. Benomyl is an antifungal agent that selectively promotes basidiomycetes growth (32). Hyphal fragments were prepared via ceramic bead-beating, and the resulting supernatant was diluted 1:10 for EUCAST and 1:50 for CLSI protocols. Microdilution plates were incubated at 35°C for 96 h before visual reading. MICs were defined as the lowest concentration yielding complete (100%) growth inhibition.

The concentration of hyphal fragments (fragments/mL) was quantified by counting on a KOVA slide prior to inoculation for 39 strains. Viability counts were performed systematically on all isolates, with colony enumeration at 48–72 h post-inoculation. Each MIC determination was conducted in duplicate on separate days; in cases of discrepancy (≥2 dilution steps), a third test was performed. *Aspergillus fumigatus* ATCC 204304 was included on each plate as a quality control strain.

## RESULTS

### Phylogenetic analysis

The concatenated alignment from multilocus sequencing of the LSU, EF1-α, and RPB2 regions comprised 2,213 base pairs (LSU: 654 bp; EF1-α: 774 bp; and RPB2: 785 bp) of which 552 bp were variable sites (LSU: 46 bp; EF1-α: 215 bp; and RPB2: 291 bp) and 283 bp were parsimony-informative sites (LSU: 7 bp; EF1-α: 88 bp; and RPB2: 188 bp).

**Fig 2:**
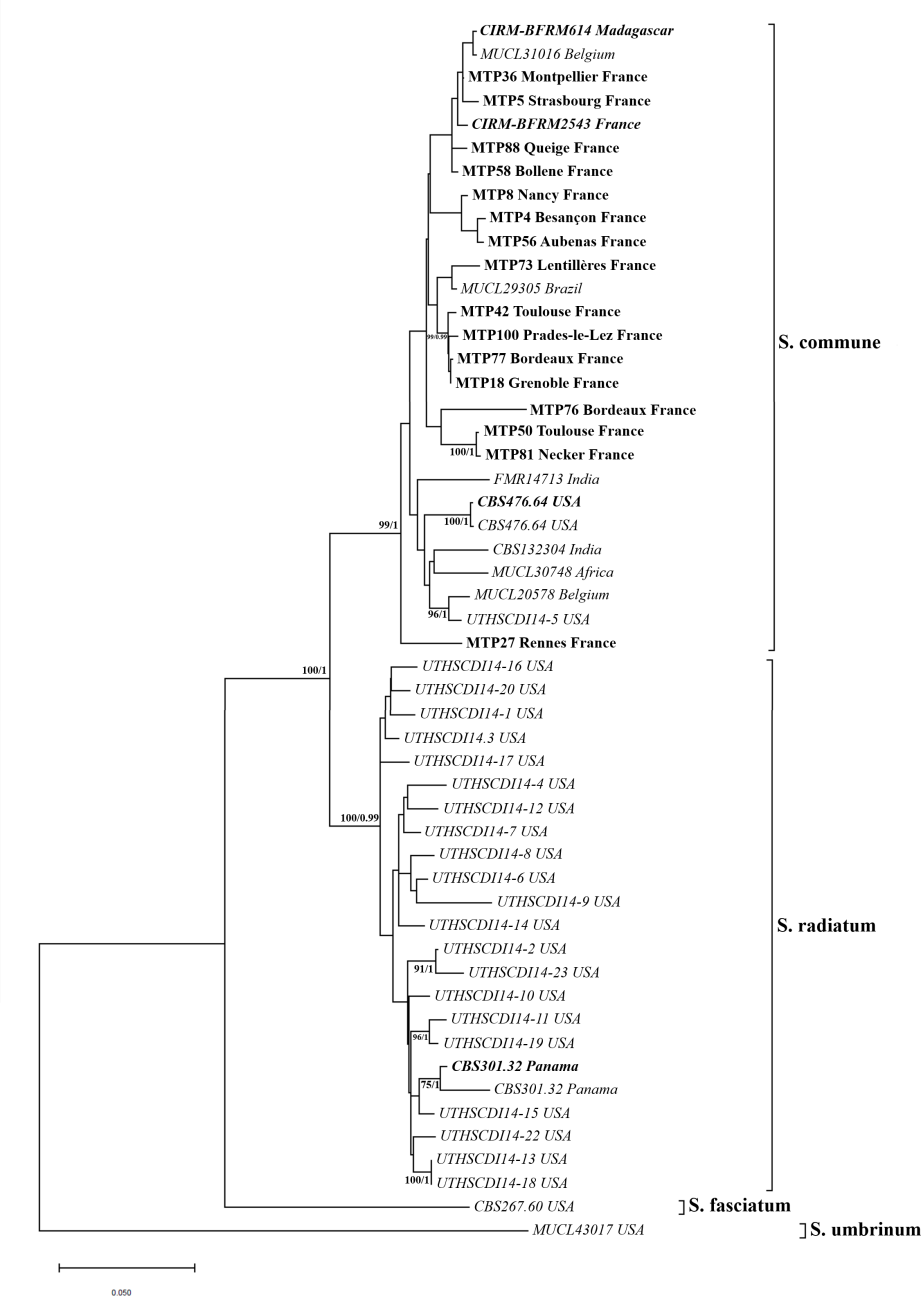
Phylogenetic tree of isolates built with maximum likelihood on Mega11 with the concatenated sequences of the LSU, EF-1α, and RPB2. Bootstrap values determined with Mega11 and posterior probability scores determined with MrBayes software were indicated when superior to 70/0.95 (bootstrap value/posterior probability score). Isolates sequenced by the authors are in bold. Sequences of non-bold isolates are from Siquiera et al. (20). Reference strains from international fungal collection are in *italic*. CBS, CBS Fungal Biodiversity Center (Westerdijk Fungal Biodiversity Institute, the Netherlands); CIRM-CF, Center International de Ressources Microbiennes – Champignons Filamenteux; EF-1α, translation elongating factor 1α ; FMR, Facultat de Medicina de Reus (Spain); LSU, large subunit portion of the ribosomal DNA; MTP, isolates collected in the purpose of this work by the Montpellier Mycology University Hospital Laboratory (France); MUCL, Université Catholique de Louvain (Belgium); RPB2, RNA polymerase II second-largest subunit; UTHSC, University of Texas Science Center (San Antonio, TX, USA).

Phylogenetic trees constructed using both ML and Bayesian inference methods yielded congruent topologies. The clades corresponding to *S. commune* and *S. radiatum* were strongly supported, with bootstrap/posterior probability values of 99/1 and 99/0.99, respectively (Fig. 2). All isolates collected for this study and from CIRM-CF collection (18/18) clustered within the *S. commune* clade.

The tree was rooted using *S. fasciatum* and *S. umbrinum*, which exhibited substantial genetic divergence from *S. commune* (8.1% and 14.73%, respectively) and *S. radiatum* (7.78% and 14.13%, respectively). The average genetic distance between *S. commune* and *S. radiatum clades* was 5.42%. Within-clade mean genetic distances were 2.56% for *S. commune* and 2.09% for *S. radiatum*.

### Antifungal susceptibility testing

Inoculum standardization was mostly effectively achieved using the bead-beating method, which yielded a mean viability count of 1,280 **±** 1,320 colony forming unit (CFU)/mL with a coefficient of variation (CV) of 103%. This was superior to both manual vortexing which yielded low CFU concentration (200 ± 160 CFU/mL, CV = 82%) that was unsuitable for antifungal susceptibility testing, and the overlaying method, which had a high CV (6,680 **±** 9,660 CFU/mL, CV = 145%) (Table S3). The selected method demonstrated high repeatability (>95% of MICs within one dilution step; Table S4) and reproducibility (>95% of MICs within two dilution steps; Table S5).

Prior to EUCAST testing, the working inoculum contained 13,564 **±** 12,193 hyphal fragments/mL across 39 strains. Viability counts averaged 1,391 **±** 1,449 CFU/mL for EUCAST and 632 **±** 626 CFU/mL for CLSI, indicating that approximately 1 in 10 hyphal fragments was capable of forming a colony.

MIC results are summarized in Table 1. AMB and VOR exhibited low geometrical mean (GM) MICs in both EUCAST (AMB: 0.39 µg/mL and VOR: 0.24 µg/mL) and CLSI (AMB: 0.1 µg/mL and VOR: 0.2 µg/mL). In contrast, TER showed high MICs (>8 µg/mL) across both methods.

**TABLE 1:** Antifungal susceptibility profiles of 113 strains of *Schizophyllum commune* in EUCAST and CLSI reference microdilution methods^*a*^

Method	Parameter	MIC (µg/mL)					
		VOR	POS	ISA	ITR	TER	AMB
EUCAST	GM	0.24	4.22	3.64	3.9	>8	0.39
	MIC50	0.125	4	2	2	>8	0.25
	MIC90	0.5	8	8	8	>8	0.5
	Range	0.03–1	0.5 to >8	0.125–16	0.016 to >8	>8 to >8	0.03–1
CLSI	GM	0.2	0.9	0.74	0.81	>8	0.1
	MIC50	0.125	1	0.5	0.5	>8	0.06
	MIC90	0.5	1	1	1	>8	0.25
	Range	0.03–1	0.03–2	0.03–4	0.03 to >8	2 to >8	0.03–0.25

^*a*^ AMB, amphotericin B; GM, geometrical mean; ISA, isavuconazole; ITR, itraconazole; MIC, minimal inhibitory concentration; POS, posaconazole; TER, terbinafine; VOR, voriconazole.

Notable discrepancies were observed for POS, ISA, and ITR, with MICs approximately two dilution steps higher in EUCAST compared to CLSI (POS: 4.22 µg/mL vs 0.9 µg/mL, ISA: 3.64 µg/mL vs 0.74 µg/mL, and ITR: 3.9 µg/mL vs 0.81 µg/mL, respectively).

Correlation analysis revealed moderate positive associations between MIC values and viability counts for POS (*ρ* = 0.36), ISA (*ρ* = 0.40), and ITR (*ρ* = 0.35). In contrast, correlations were weak for VOR (*ρ* = 0.11) and TER (*ρ* = 0.14), and no significant correlation was observed for AMB (Table 2).

**TABLE 2:** Pearson correlation coefficient between MIC values and the viability counts in EUCAST and CLSI methods^*a*^^*,^b^*^

	VOR	POS	ISA	ITR	TER	AMB
*P* value	**0.005316**	**<2.2e−16**	**<2.2e−16**	**<2.2e−16**	**4.357e−08**	0.2638
Pearson’s coefficient	**0.1118121**	**0.3609811**	**0.4009678**	**0.3526957**	**0.1402592**	0.04465783
*n*	620	614	617	621	630	629

^*a*^ AMB, amphotericin B; ISA, isavuconazole; ITR, itraconazole; POS, posaconazole; TER, terbinafine; VOR, voriconazole.

^*b*^ Statistically significant *P* values and Pearson coefficients have been highlighted in bold.

The single *S. radiatum* strain tested (CBS301.32) did not exhibit a susceptibility profile distinct from the other *S. commune* isolates (Table S2).

## DISCUSSION

This study evaluated the antifungal susceptibility profiles of 113 isolates against six agents, representing the first investigation to include such a substantial number of isolates derived from both human clinical specimens and environmental sources. Antifungal susceptibility testing of *S. commune* remains a methodological challenge, primarily due to the absence of standardized protocols. To date, only three studies have evaluated five or more isolates (up to 30) using modified CLSI procedures (31). In this study, the modifications to the EUCAST/CLSI methods included: (i) growing the isolates on a Sabouraud dextrose agar plate supplemented with 10 mg/L of benomyl for 7–10 days at 30°C; (ii) using an inoculum obtained after hyphal fragmentation and homogenization by bead beating of 1 cm^2^ piece of culture. (iii) Microdilution plates were incubated at 35°C for 96 h. (iv) MIC endpoints were read visually at 100% of inhibition.

In this work, the lowest GM MICs were observed for VOR and AMB, with values of 0.24 and 0.39 µg/mL in EUCAST, and 0.2 and 0.1 µg/mL in CLSI, respectively. These findings were consistent with previous reports using adapted CLSI methodologies: Chowdhary et al. (20) reported MICs of 0.24 µg/mL (VOR) and 0.29 µg/mL (AMB); González et al. (21) reported 0.57 and 0.5 µg/mL; and Siqueira et al. (22) reported 0.20 and 0.09 µg/mL, respectively.

TER exhibited the highest MICs (>8 µg/mL) in both EUCAST and CLSI methods, which contrasts with the only prior study evaluating TER susceptibility (22), where a GM MIC of 0.79 µg/mL was reported, with a wide range from 0.03 to 16 µg/mL. It is important to note that TER MIC endpoints in that study were read at 80% inhibition, whereas our study used a stricter 100% inhibition criterion as recommended by EUCAST and CLSI protocols.

For the remaining azoles (POS, ISA, and ITR), MIC_50_ values obtained via EUCAST were consistently two dilution steps higher than those obtained via CLSI (POS: 4 µg/mL vs.1 µg/mL, ISA: 2 µg/mL vs 0.5 µg/mL, and ITR: 2 µg/mL vs 0.5 µg/mL). Previous studies using adapted CLSI methods reported low GM MICs for these agents: POS (0.11 µg/mL [20], 0.43 µg/mL [21], and 0.20 µg/mL [22]), ISA (0.19 µg/mL [20]), and ITR (0.20 µg/mL [20], 0.06 µg/mL [21], and 0.37 µg/mL [22]). These discrepancies may be partially attributed to differences in inoculum preparation between EUCAST and CLSI protocols. Specifically, the initial hyphal fragment suspension is diluted 1:10 in EUCAST versus 1:50 in CLSI, resulting in higher viability counts in EUCAST (1,391 ± 1,449 CFU/mL) compared to CLSI (632 ± 626 CFU/mL). This suggests that lower inoculum concentrations may yield lower MICs for POS, ISA, and ITR. This hypothesis is supported by the observed positive correlations between MICs and viability counts for these agents (*ρ* = 0.36, 0.40, and 0.35, respectively), in contrast to weaker or non-significant correlations for VOR, TER, and AMB (*ρ* = 0.11, 0.14, and 0.04, respectively).

Previous studies have inconsistently reported inoculum concentrations as either hyphal fragments/mL (20, 22) or CFU/mL (21). Chowdhary et al. (20) and Siqueira et al. (22) used inocula of 25,000–50,000 hyphal fragments/mL, adapted from the CLSI M38 document (31) for nondermatophyte molds, which recommend 4,000–50,000 CFU/mL. González et al. (21) employed a macrobroth dilution method targeting 10,000 CFU/mL. Our findings demonstrate that hyphal fragment/mL and CFU/mL are not equivalent for *S. commune*, as only approximately 1 in 10 hyphal fragments forms a colony. Therefore, a target inoculum of 1,000–3,000 CFU/mL, as recommended for dermatophytes in CLSI M38 (31), appears more appropriate and aligns with our observed viability counts.

Another potential source of MIC variability is the misidentification of *S. commune* with its sibling species *S. radiatum,* which has been reported to exhibit higher MICs, particularly for POS and ITR (22). Using the same multilocus phylogenetic approach (LSU, EF-1α, and RPB2) as Siqueira et al. (22), we confirmed that all 18 sequenced isolates belonged to *S. commune*, with no *S. radiatum* identified. The single *S. radiatum* strain (CBS301.32) tested in this study displayed a susceptibility profile comparable to *S. commune* isolates (Table S2).

This study presents several limitations: only 20 out of 113 isolates were characterized for the EF-1α and RPB2 genes due to cost and time constraints; antifungal susceptibility testing was conducted at a single center, preventing inter-laboratory comparisons and the establishment of epidemiological cut-off values. It nonetheless remains the most extensive investigation conducted to date on this basidiomycete.

In conclusion, our data demonstrate that isolates included in this study are identified as *S. commune* rather than *S. radiatum*. The standardization and adaptation of EUCAST and CLSI protocols—particularly regarding inoculum preparation—are essential to ensure accurate antifungal susceptibility testing of this non-sporulating mold. Further work could evaluate the possibility of applying this method to other non-sporulating basidiomycetes. Among the agents tested, AMB and VOR exhibited the highest *in vitro* activity, in alignment with current therapeutic recommendations (6).

## SUPPLEMENTAL MATERIAL

Tables S1 to S5 jcm.01442-25-s0001.docx Characteristics of the 113 isolates of *Schizophyllum commune* and additional technical results.

ASM does not own the copyrights to Supplemental Material that may be linked to, or accessed through, an article. The authors have granted ASM a non-exclusive, world-wide license to publish the Supplemental Material files. Please contact the corresponding author directly for reuse.
