# Supplementary material for: Antifungal susceptibility and molecular characterization of clinical and environmental isolates of Schizophyllum commune
Source: J Clin Microbiol. 2026 Feb 18;64(3):e01442-25. doi: 10.1128/jcm.01442-25 (PMC12977503; doi:10.1128/jcm.01442-25)
Supplement: Tables S1 to S5 — Characteristics of the 113 isolates of Schizophyllum commune and additional technical results. [file jcm.01442-25-s0001.docx]

**Table S1:** Sequences of the primers specifically designed for the translation elongating factor 1α (EF-1α) and RNA polymerase II second-largest subunit (RPB2) in *S. commune.*

| **Gene** | **Primers' position** | **Forward/Reverse** | **Sequences** |
| --- | --- | --- | --- |
| **EF-1 α** | external | Forward | TCTCCTTGCCTTCACCCTCG |
|  |  | Reverse | TCCTTGACGGAGACGTTCCTG |
|  | internal | Forward | GAGGACCGTTTCAACGAAATC |
|  |  | Reverse | TGCATCTCGACGGACTTGACC |
| **RPB2** | external | Forward | TACCGCTACTTGCAGAAGGTA |
|  |  | Reverse | TAACCARCGAGTCCCACTTCG |
|  | internal | Forward | GTCTCGTCAAGAACCTCTCGCTC |
|  |  | Reverse | TCCTGCAAGAAGTCGATGACG |

**Table S2:** Characteristics of the 113 isolates of *Schizophyllum commune* included in the study with corresponding MICs and GenBank accession numbers.for LSU and if available for EF-1α and RPB2.

EF-1α, translation elongating factor 1α; LSU, large sub-unit portion of the ribosomal DNA; MIC, minimal inhibitory concentration, RPB2, RNA polymerase II second-largest subunit

|  |  |  |  | **GenBank accession no.** | | | **EUCAST** | | | | | | **CLSI** | | | | | |
| --- | --- | --- | --- | --- | --- | --- | --- | --- | --- | --- | --- | --- | --- | --- | --- | --- | --- | --- |
| **Strain** | **Origin** | **Geographical origin** | **Sample type** | ***LSU*** | ***EF-1α*** | ***RPB2*** | **VOR** | **POS** | **ISA** | **ITR** | **TER** | **AMB** | **VOR** | **POS** | **ISA** | **ITR** | **TER** | **AMB** |
| **MTP1** | patient | Universitary hospital of Nimes, France | Brochio-alveolar lavage fluid | PX210936 |  |  | 0.25 | 0.5 | 0.5 | 1 | >8 | 0.06 | 0.06 | 0.5 | 0.25 | 0.5 | 8 | 0.125 |
| **MTP2** | patient | University hospital of La Pitié-Salpetriere, Paris, France | Sinus sample | PX210937 |  |  | 0.5 | 4 | 4 | 2 | >8 | 0.5 | 0.25 | 1 | 1 | 1 | >8 | 0.06 |
| **MTP3** | patient | Universitary hospital of Strasbourg, France | Brochio-alveolar lavage fluid | PX210938 |  |  | 0.06 | 2 | 1 | 1 | >8 | 0.125 | 0.25 | 2 | 2 | 1 | >8 | 0.125 |
| **MTP4** | patient | University hospital of Besançon, France | Bronchial aspirate | PX210939 | PX278096 | PX278116 | 0.125 | 1 | 2 | 1 | >8 | 0.25 | 0.06 | 0.5 | 0.25 | 0.25 | >8 | 0.125 |
| **MTP5** | patient | Universitary hospital of Strasbourg, France | Tracheal aspirate | PX210940 | PX278097 | PX278117 | 0.125 | 1 | 2 | 1 | >8 | 0.125 | 0.25 | 1 | 0.5 | 0.5 | >8 | 0.125 |
| **MTP6** | patient | Universitary hospital of Nice, France | Sinus sample | PX210941 |  |  | 0.25 | 4 | 4 | 2 | >8 | 0.5 | 0.25 | 0.5 | 1 | 0.5 | >8 | 0.125 |
| **MTP7** | patient | Universitary hospital of Nice, France | Sinus sample | PX210942 |  |  | 0.25 | 4 | 4 | 4 | >8 | 0.25 | 0.125 | 0.5 | 0.5 | 0.5 | >8 | 0.06 |
| **MTP8** | patient | Universitary hospital of Nancy, France | Sinus sample | PX210943 | PX278099 | PX278119 | 1 | >8 | 8 | >8 | >8 | 0.25 | 0.25 | 2 | 1 | 1 | >8 | 0.125 |
| **MTP9** | patient | Universitary hospital of Nancy, France | Sinus sample | PX210944 |  |  | 0.125 | 1 | 2 | 1 | >8 | 0.25 | 0.03 | 0.06 | 0.03 | 0.03 | 8 | 0.03 |
| **MTP10** | patient | Universitary hospital of Nice, France | Brochio-alveolar lavage fluid | PX210945 |  |  | 0.5 | >8 | 16 | 4 | >8 | 0.25 | 0.5 | 2 | 4 | 2 | >8 | 0.125 |
| **MTP11** | patient | Universitary hospital of Nice, France | Bronchial aspirate | PX210946 |  |  | 0.5 | 8 | 16 | 8 | >8 | 0.5 | 0.5 | 1 | 2 | 1 | >8 | 0.125 |
| **MTP12** | patient | Universitary hospital of Nimes, France | Sinus sample | PX210947 |  |  | 0.25 | 8 | 4 | >8 | >8 | 0.5 | 0.125 | 1 | 1 | 1 | >8 | 0.06 |
| **MTP13** | patient | Universitary hospital of Montpellier, France | Unknown | PX210948 |  |  | 0.25 | 4 | 2 | 2 | >8 | 0.06 | 0.06 | 1 | 0.5 | 0.5 | >8 | 0.03 |
| **MTP14** | patient | Universitary hospital of Grenoble, France | Bronchial aspirate | PX210949 |  |  | 0.25 | 4 | 8 | 2 | >8 | 0.06 | 0.25 | 1 | 1 | 0.5 | >8 | 0.25 |
| **MTP15** | patient | Universitary hospital of Grenoble, France | Unknown | PX210950 |  |  | 0.25 | 2 | 2 | 2 | >8 | 0.25 | 0.25 | 1 | 1 | 0.5 | >8 | 0.25 |
| **MTP16** | patient | Universitary hospital of Nice, France | Unknown | PX210951 |  |  | 0.06 | 1 | 0.5 | 0.5 | >8 | 0.25 | 0.06 | 0.5 | 0.125 | 0.06 | 8 | 0.06 |
| **MTP17** | patient | Universitary hospital of Nimes, France | Unknown | PX210952 |  |  | 0.25 | 4 | 4 | 1 | >8 | 0.25 | 0.125 | 0.5 | 0.5 | 0.5 | >8 | 0.06 |
| **MTP18** | patient | Universitary hospital of Grenoble, France | Sinus sample | PX210953 | PX278107 | PX278127 | 0.125 | 2 | 2 | 2 | >8 | 0.25 | 0.03 | 0.5 | 0.25 | 0.25 | 8 | 0.125 |
| **MTP19** | patient | Universitary hospital of Grenoble, France | Sinus sample | PX210954 |  |  | 0.125 | 4 | 2 | 1 | >8 | 0.06 | 0.125 | 0.5 | 0.5 | 0.25 | >8 | 0.25 |
| **MTP20** | patient | Universitary hospital of Grenoble, France | Sinus sample | PX210955 |  |  | 0.125 | 4 | 2 | 1 | >8 | 0.25 | 0.125 | 1 | 1 | 1 | >8 | 0.125 |
| **MTP21** | patient | Universitary hospital of Montpellier, France | Unknown | PX210956 |  |  | 0.125 | 2 | 2 | 1 | >8 | 0.25 | 0.03 | 0.03 | 0.06 | 0.125 | 4 | 0.06 |
| **MTP22** | patient | Universitary hospital of Montpellier, France | Unknown | PX210957 |  |  | 0.25 | 8 | 4 | 1 | >8 | 0.125 | 0.125 | 0.5 | 0.5 | 0.06 | >8 | 0.06 |
| **MTP23** | patient | Universitary hospital of Grenoble, France | Sinus sample | PX210958 |  |  | 0.125 | 4 | 4 | 4 | >8 | 0.06 | 0.25 | 2 | 1 | 2 | >8 | 0.25 |
| **MTP24** | patient | France | Unknown | PX210959 |  |  | 0.125 | 2 | 2 | 0.016 | >8 | 0.25 | 0.25 | 0.5 | 0.5 | 0.25 | >8 | 0.125 |
| **MTP25** | patient | Universitary hospital of Grenoble, France | Expectoration | PX210960 |  |  | 0.06 | 1 | 1 | 0.5 | >8 | 0.25 | 0.06 | 0.25 | 0.06 | 0.06 | 4 | 0.125 |
| **MTP26** | patient | Universitary hospital of Grenoble, France | Sinus sample | PX210961 |  |  | 0.125 | 4 | 2 | 2 | >8 | 0.25 | 0.03 | 0.25 | 0.125 | 0.06 | 8 | 0.25 |
| **MTP27** | patient | Universitary hospital of Rennes, France | Expectoration | PX210962 | PX278094 | PX278114 | 0.5 | 1 | 2 | 1 | >8 | 0.125 | 0.125 | 0.5 | 1 | 1 | >8 | 0.125 |
| **MTP28** | patient | Universitary hospital of Nice, France | Unknown | PX210963 |  |  | 0.06 | 1 | 0.5 | 2 | >8 | 0.06 | 0.06 | 0.5 | 0.25 | 0.5 | >8 | 0.125 |
| **MTP29** | patient | Universitary hospital of Montpellier, France | Sinus sample | PX210964 |  |  | 0.25 | 2 | 4 | 1 | >8 | 0.25 | 0.25 | 1 | 1 | 2 | >8 | 0.125 |
| **MTP30** | patient | Universitary hospital of Montpellier, France | Sinus sample | PX210965 |  |  | 0.125 | 4 | 8 | 2 | >8 | 0.25 | 0.25 | 1 | 0.5 | 0.5 | >8 | 0.06 |
| **MTP31** | patient | Universitary hospital of Montpellier, France | Sinus sample | PX210966 |  |  | 0.25 | 8 | 4 | 1 | >8 | 0.25 | 0.5 | 1 | 0.5 | 1 | >8 | 0.03 |
| **MTP32** | patient | Universitary hospital of Montpellier, France | Sinus sample | PX210967 |  |  | 0.25 | 4 | 2 | 4 | >8 | 0.125 | 0.125 | 1 | 0.25 | 0.5 | >8 | 0.06 |
| **MTP33** | patient | Universitary hospital of Montpellier, France | Sinus sample | PX210968 |  |  | 0.5 | 4 | 2 | 4 | >8 | 0.125 | 0.25 | 1 | 1 | 0.5 | >8 | 0.06 |
| **MTP34** | patient | Universitary hospital of Montpellier, France | Expectoration | PX210969 |  |  | 0.25 | 2 | 1 | 1 | >8 | 0.125 | 0.5 | 1 | 1 | 1 | >8 | 0.06 |
| **MTP35** | patient | Universitary hospital of Montpellier, France | Sinus sample | PX210970 |  |  | 0.25 | 4 | 4 | 1 | >8 | 0.06 | 0.25 | 1 | 1 | 0.5 | >8 | 0.03 |
| **MTP36** | patient | Universitary hospital of Montpellier, France | Sinus sample | PX210971 | PX278098 | PX278118 | 0.5 | 2 | 4 | 2 | >8 | 0.25 | 0.06 | 0.25 | 0.06 | 0.125 | 2 | 0.03 |
| **MTP37** | patient | Universitary hospital of Montpellier, France | Sinus sample | PX210972 |  |  | 0.25 | 2 | 2 | 1 | >8 | 0.5 | 0.06 | 0.5 | 0.25 | 0.25 | >8 | 0.03 |
| **MTP38** | patient | Universitary hospital of Montpellier, France | Sinus sample | PX210973 |  |  | 1 | >8 | 16 | 8 | >8 | 0.25 | 0.25 | 1 | 0.5 | 0.25 | >8 | 0.03 |
| **MTP39** | patient | Universitary hospital of Toulouse, France | Brochio-alveolar lavage fluid | PX210974 |  |  | 0.125 | 2 | 4 | 1 | >8 | 0.5 | 0.25 | 1 | 2 | 1 | >8 | 0.03 |
| **MTP40** | patient | Universitary hospital of Toulouse, France | Sinus sample | PX210975 |  |  | 0.125 | 2 | 1 | 2 | >8 | 0.25 | 0.125 | 1 | 1 | 1 | >8 | 0.125 |
| **MTP41** | patient | Universitary hospital of Toulouse, France | Sinus sample | PX210976 |  |  | 0.125 | 1 | 2 | 1 | >8 | 0.25 | 0.06 | 0.5 | 0.5 | 0.25 | >8 | 0.125 |
| **MTP42** | patient | Universitary hospital of Toulouse, France | Tracheal aspirate | PX210977 | PX278100 | PX278120 | 0.125 | 4 | 2 | 4 | >8 | 0.5 | 0.25 | 1 | 1 | 1 | >8 | 0.125 |
| **MTP43** | patient | Universitary hospital of Toulouse, France | Brochio-alveolar lavage fluid | PX210978 |  |  | 0.25 | 8 | 4 | 8 | >8 | 0.25 | 0.125 | 1 | 0.5 | 1 | >8 | 0.25 |
| **MTP44** | patient | Universitary hospital of Toulouse, France | Expectoration | PX210979 |  |  | 0.125 | 2 | 2 | 4 | >8 | 0.25 | 0.125 | 0.5 | 0.5 | 0.5 | >8 | 0.125 |
| **MTP45** | patient | Universitary hospital of Toulouse, France | Sinus sample | PX210980 |  |  | 0.125 | 1 | 1 | 2 | >8 | 0.5 | 0.125 | 1 | 1 | 1 | >8 | 0.125 |
| **MTP46** | patient | Universitary hospital of Toulouse, France | Brochio-alveolar lavage fluid | PX210981 |  |  | 0.125 | 8 | 4 | >8 | >8 | 0.25 | 0.125 | 2 | 0.25 | 0.5 | >8 | 0.125 |
| **MTP47** | patient | Universitary hospital of Toulouse, France | Expectoration | PX210982 |  |  | 0.25 | 2 | 8 | 1 | >8 | 0.5 | 0.25 | 1 | 2 | 1 | >8 | 0.06 |
| **MTP48** | patient | Universitary hospital of Toulouse, France | Sinus sample | PX210983 |  |  | 0.06 | 0.5 | 1 | 1 | >8 | 0.25 | 0.06 | 1 | 0.125 | 0.125 | 8 | 0.06 |
| **MTP49** | patient | Universitary hospital of Toulouse, France | Expectoration | PX210984 |  |  | 0.125 | 2 | 4 | 2 | >8 | 0.5 | 1 | 1 | 2 | 2 | >8 | 0.06 |
| **MTP50** | patient | Universitary hospital of Toulouse, France | Sinus sample | PX210985 | PX278101 | PX278121 | 0.25 | 4 | 1 | 2 | >8 | 0.03 | 0.25 | 0.5 | 1 | 0.25 | >8 | 0.03 |
| **MTP51** | patient | Universitary hospital of Toulouse, France | Sinus sample | PX210986 |  |  | 0.25 | 4 | 2 | 4 | >8 | 0.125 | 0.25 | 1 | 1 | 4 | >8 | 0.06 |
| **MTP52** | patient | Universitary hospital of Toulouse, France | Bronchial aspirate | PX210987 |  |  | 0.06 | 0.5 | 1 | 1 | >8 | 0.125 | 0.06 | 1 | 0.125 | 0.25 | >8 | 0.06 |
| **MTP53** | patient | Universitary hospital of Toulouse, France | Brochio-alveolar lavage fluid | PX210988 |  |  | 0.25 | 1 | 0.5 | 4 | >8 | 0.125 | 0.25 | 0.5 | 1 | 0.5 | >8 | 0.03 |
| **MTP54** | patient | Universitary hospital of Toulouse, France | Sinus sample | PX210989 |  |  | 0.25 | 4 | 2 | 8 | >8 | 0.125 | 0.25 | 1 | 0.5 | 0.5 | >8 | 0.03 |
| **MTP55** | environnement | Ano Poroia, Central macedonia, Greece | wood | PX210990 |  |  | 0.5 | 8 | 8 | >8 | >8 | 0.25 | 0.25 | 1 | 1 | 0.5 | >8 | 0.25 |
| **MTP56** | environnement | Aubenas, Ardeche, France | wood | PX210991 | PX278102 | PX278122 | 0.125 | 2 | 4 | >8 | >8 | 0.5 | 0.125 | 1 | 1 | 0.5 | >8 | 0.03 |
| **CIRM-BFRM 614** | environnement | Madagascar | wood | PX210992 | PX278104 | PX278124 | 0.03 | 1 | 0.125 | 1 | >8 | 0.25 | 0.125 | 0.5 | 0.25 | 0.25 | >8 | 0.03 |
| **MTP58** | environnement | Bollene, Vaucluse, France | wood | PX210993 | PX278103 | PX278123 | 0.125 | 4 | 2 | 2 | >8 | 1 | 0.125 | 0.5 | 0.25 | 0.25 | >8 | 0.06 |
| **CBS 301.32 *S. radiatum*** | environnement | Panama | wood | PX210994 | PX278112 | PX278132 | 0.125 | 1 | 4 | 1 | >8 | 0.25 | 0.25 | 0.125 | 0.25 | 0.125 | 4 | 0.06 |
| **CBS 476.64 *S. commune*** | environnement | USA | wood | PX210995 | PX278106 | PX278126 | 0.06 | 0.5 | 0.5 | 0.5 | >8 | 0.125 | 0.06 | 0.06 | 0.25 | 0.06 | >8 | 0.03 |
| **MTP61** | environnement | Montpellier, Herault, France | wood | PX210996 |  |  | 0.125 | 4 | 2 | 2 | >8 | 0.5 | 0.125 | 1 | 0.5 | 0.5 | >8 | 0.03 |
| **MTP62** | environnement | Montpellier, Herault, France | wood | PX210997 |  |  | 0.125 | 4 | 2 | 4 | >8 | 0.5 | 0.125 | 0.5 | 0.5 | 0.5 | >8 | 0.25 |
| **CIRCM-BFRM 1860** | environnement | La Bouilladisse, Bouches du Rhone, France | wood | PX210998 |  |  | 0.25 | 8 | 1 | 2 | >8 | 1 | 0.25 | 1 | 0.5 | 0.25 | >8 | 0.125 |
| **CIRCM-BFRM 2543** | environnement | France | wood | PX210999 | PX278105 | PX278125 | 0.25 | 4 | 4 | 2 | >8 | 0.5 | 0.25 | 1 | 0.5 | 2 | >8 | 0.25 |
| **CIRCM-BFRM 823** | environnement | Isere, France | wood | PX211000 |  |  | 0.06 | 0.5 | 0.25 | 1 | >8 | 0.5 | 0.125 | 1 | 0.5 | 0.5 | >8 | 0.25 |
| **CIRCM-BFRM 861** | environnement | Freydieres, Isere, France | wood | PX211001 |  |  | 0.125 | 1 | 0.5 | 1 | >8 | 1 | 0.125 | 1 | 0.25 | 1 | >8 | 0.125 |
| **CIRCM-BFRM 862** | environnement | Villard de Lans, Isere, France | wood | PX211002 |  |  | 0.5 | 4 | 4 | 1 | >8 | 0.5 | 0.5 | 2 | 1 | 1 | >8 | 0.125 |
| **MTP68** | environnement | Portet-sur-Garonne, Haute-Garonne, France | wood | PX211003 |  |  | 0.125 | 4 | 2 | 2 | >8 | 1 | 0.25 | 2 | 1 | 1 | >8 | 0.25 |
| **MTP69** | environnement | Montpellier, Herault, France | wood | PX211004 |  |  | 0.125 | 4 | 1 | 2 | >8 | 0.5 | 0.25 | 1 | 1 | 1 | >8 | 0.25 |
| **MTP70** | environnement | Montpellier, Herault, France | wood | PX211005 |  |  | 0.125 | 4 | 4 | 4 | >8 | 0.5 | 0.5 | 1 | 0.5 | 1 | >8 | 0.25 |
| **MTP71** | environnement | Montpellier, Herault, France | wood | PX211006 |  |  | 0.125 | 2 | 1 | 8 | >8 | 0.5 | 0.125 | 1 | 0.25 | 0.5 | >8 | 0.125 |
| **MTP72** | environnement | Le Bourget-du-Lac, Savoie, France | wood | PX211007 |  |  | 0.125 | 1 | 2 | 2 | >8 | 1 | 0.03 | 0.25 | 0.5 | 0.125 | 8 | 0.125 |
| **MTP73** | environnement | Lentillères, Ardeche, France | wood | PX211008 | PX278108 | PX278128 | 0.125 | 4 | 1 | 4 | >8 | 0.5 | 0.125 | 1 | 0.5 | 0.5 | >8 | 0.125 |
| **MTP74** | environnement | Lespinassiere, Aude, France | wood | PX211009 |  |  | 1 | >8 | 8 | >8 | >8 | 0.5 | 0.25 | 1 | 2 | 1 | >8 | 0.125 |
| **MTP75** | environnement | Lespinassiere, Aude, France | wood | PX211010 |  |  | 0.5 | 4 | 4 | 4 | >8 | 0.25 | 0.25 | 1 | 0.5 | 1 | >8 | 0.125 |
| **MTP76** | patient | University hospital of Bordeaux, France | Sinus sample | PX211011 | PX278095 | PX278115 | 0.25 | 4 | 1 | 2 | >8 | 0.25 | 0.25 | 1 | 0.5 | 0.5 | >8 | 0.06 |
| **MTP77** | patient | University hospital of Bordeaux, France | Sinus sample | PX211012 | PX278093 | PX278113 | 0.5 | 8 | 8 | 8 | >8 | 0.06 | 0.5 | 2 | 2 | >8 | >8 | 0.125 |
| **MTP78** | patient | University hospital of Bordeaux, France | Sinus sample | PX211013 |  |  | 0.25 | 4 | 1 | 4 | >8 | 0.03 | 0.125 | 1 | 0.5 | 0.5 | >8 | 0.03 |
| **MTP79** | patient | University hospital of Bordeaux, France | Unknown | PX211014 |  |  | 0.25 | 4 | 4 | 2 | >8 | 0.25 | 0.125 | 1 | 0.5 | 0.5 | >8 | 0.06 |
| **MTP80** | patient | University hospital of Bordeaux, France | Unknown | PX211015 |  |  | 0.25 | 4 | 4 | 2 | >8 | 1 | 0.125 | 1 | 0.5 | 2 | >8 | 0.06 |
| **MTP81** | patient | University hospital of Necker, Paris, France | Sinus sample | PX211016 | PX278109 | PX278129 | 0.125 | 2 | 0.5 | 1 | >8 | 0.25 | 0.06 | 0.5 | 0.25 | 0.125 | >8 | 0.03 |
| **MTP82** | patient | University hospital of Necker, Paris, France | Sinus sample | PX211017 |  |  | 0.25 | 2 | 4 | 1 | >8 | 0.5 | 0.125 | 0.5 | 1 | 0.5 | >8 | 0.03 |
| **MTP83** | patient | Universitary hospital of Toulouse, France | Sinus sample | PX211018 |  |  | 0.25 | 4 | 1 | 2 | >8 | 0.5 | 0.25 | 1 | 0.5 | 0.5 | >8 | 0.03 |
| **MTP84** | patient | Universitary hospital of Toulouse, France | Sinus sample | PX211019 |  |  | 0.125 | 8 | 2 | 4 | >8 | 0.25 | 0.125 | 1 | 0.25 | 0.25 | >8 | 0.03 |
| **MTP85** | patient | Universitary hospital of Toulouse, France | Sinus sample | PX211020 |  |  | 0.06 | 2 | 2 | 2 | >8 | 0.5 | 0.06 | 0.25 | 0.5 | 0.125 | 8 | 0.03 |
| **MTP86** | patient | Universitary hospital of Toulouse, France | Sinus sample | PX211021 |  |  | 0.125 | 4 | 2 | 2 | >8 | 0.25 | 0.06 | 0.125 | 0.125 | 0.125 | 4 | 0.06 |
| **MTP87** | patient | Universitary hospital of Toulouse, France | Sinus sample | PX211022 |  |  | 0.5 | 8 | 8 | 8 | >8 | 0.25 | 0.25 | 1 | 1 | 0.5 | >8 | 0.03 |
| **MTP88** | environnement | Queige, Savoie, France | wood | PX211023 | PX278110 | PX278130 | 0.125 | 4 | 1 | 2 | >8 | 0.5 | 0.125 | 0.5 | 0.5 | 0.25 | 8 | 0.03 |
| **MTP89** | environnement | Queige, Savoie, France | wood | PX211024 |  |  | 0.25 | 8 | 8 | 4 | >8 | 0.5 | 0.125 | 1 | 0.5 | 0.5 | >8 | 0.03 |
| **MTP90** | environnement | Queige, Savoie, France | wood | PX211025 |  |  | 0.125 | 2 | 4 | 2 | >8 | 1 | 0.125 | 1 | 0.5 | 0.5 | >8 | 0.03 |
| **MTP91** | environnement | Queige, Savoie, France | wood | PX211026 |  |  | 0.25 | 8 | 4 | 4 | >8 | 0.5 | 0.125 | 1 | 0.5 | 0.5 | >8 | 0.03 |
| **MTP92** | environnement | Queige, Savoie, France | wood | PX211027 |  |  | 0.125 | 8 | 4 | 8 | >8 | 0.5 | 0.125 | 0.5 | 0.5 | 0.5 | 8 | 0.06 |
| **MTP93** | environnement | Queige, Savoie, France | wood | PX211028 |  |  | 0.5 | 8 | 8 | 8 | >8 | 1 | 0.125 | 1 | 1 | 1 | >8 | 0.125 |
| **MTP94** | environnement | Queige, Savoie, France | wood | PX211029 |  |  | 1 | 8 | 8 | 4 | >8 | 0.5 | 0.25 | 2 | 1 | 1 | >8 | 0.125 |
| **MTP95** | environnement | Queige, Savoie, France | wood | PX211030 |  |  | 0.125 | 8 | 4 | 4 | >8 | 1 | 0.25 | 1 | 1 | 0.5 | >8 | 0.125 |
| **MTP96** | environnement | Queige, Savoie, France | wood | PX211031 |  |  | 0.125 | 4 | 1 | 1 | >8 | 0.5 | 0.125 | 1 | 0.5 | 0.5 | >8 | 0.125 |
| **MTP97** | environnement | Queige, Savoie, France | wood | PX211032 |  |  | 0.06 | 4 | 2 | 1 | >8 | 1 | 0.125 | 1 | 0.25 | 0.5 | >8 | 0.06 |
| **MTP98** | environnement | Queige, Savoie, France | wood | PX211033 |  |  | 0.125 | 4 | 4 | 4 | >8 | 1 | 0.06 | 0.5 | 0.5 | 0.5 | 8 | 0.03 |
| **MTP99** | environnement | Prades-le-lez, Herault, France | wood | PX211034 |  |  | 0.125 | 4 | 2 | 4 | >8 | 0.5 | 0.06 | 0.25 | 0.125 | 0.25 | 8 | 0.03 |
| **MTP100** | environnement | Prades-le-lez, Herault, France | wood | PX211035 | PX278111 | PX278131 | 0.125 | 4 | 8 | 2 | >8 | 0.5 | 0.125 | 0.5 | 0.5 | 0.5 | 8 | 0.06 |
| **MTP101** | environnement | Prades-le-lez, Herault, France | wood | PX211036 |  |  | 0.125 | 4 | 4 | 8 | >8 | 0.5 | 0.25 | 1 | 0.5 | 0.5 | >8 | 0.06 |
| **MTP102** | environnement | St-Clément de rivière, Herault, France | wood | PX211037 |  |  | 0.125 | 4 | 2 | 2 | >8 | 0.5 | 0.125 | 1 | 1 | 0.5 | >8 | 0.06 |
| **MTP103** | environnement | Le Lherm, Haute-Garonne, France | wood | PX211038 |  |  | 0.06 | 4 | 1 | 2 | >8 | 0.5 | 0.5 | 1 | 1 | 1 | >8 | 0.125 |
| **MTP104** | patient | Universitary hospital of Montpellier, France | Sinus sample | PX211039 |  |  | 1 | 4 | 8 | >8 | >8 | 0.25 | 0.5 | 1 | 2 | 1 | >8 | 0.06 |
| **MTP105** | patient | Universitary hospital of Nice, France | Expectoration | PX211040 |  |  | 0.5 | 8 | 8 | 8 | >8 | 0.5 | 0.25 | 2 | 1 | 2 | >8 | 0.06 |
| **MTP106** | patient | University hospital of Necker, Paris, France | Expectoration | PX211041 |  |  | 0.25 | 2 | 8 | 1 | >8 | 0.5 | 0.5 | 1 | 1 | 0.5 | >8 | 0.06 |
| **MTP107** | patient | University hospital of Besançon, France | Tracheal aspirate | PX211042 |  |  | 0.5 | 4 | 8 | 4 | >8 | 0.5 | 0.5 | 1 | 1 | 2 | >8 | 0.06 |
| **MTP108** | patient | University hospital of Besançon, France | Otorrhoea aspirate | PX211043 |  |  | 0.25 | 8 | 8 | 8 | >8 | 0.5 | 0.25 | 0.5 | 1 | 0.5 | >8 | 0.06 |
| **MTP109** | patient | University hospital of Besançon, France | Expectoration | PX211044 |  |  | 0.125 | 2 | 4 | 4 | >8 | 0.5 | 0.125 | 1 | 0.5 | 0.5 | >8 | 0.03 |
| **MTP110** | patient | University hospital of Besançon, France | Sinus sample | PX211045 |  |  | 0.25 | 4 | 8 | 8 | >8 | 0.5 | 0.5 | 2 | 1 | 1 | >8 | 0.03 |
| **MTP111** | patient | University hospital of Besançon, France | Bronchial aspirate | PX211046 |  |  | 0.25 | 4 | 8 | 4 | >8 | 0.5 | 0.25 | 1 | 2 | 1 | >8 | 0.06 |
| **MTP112** | environnement | saint romain de lerps, Ardeche, France | wood | PX211047 |  |  | 0.25 | 8 | 2 | >8 | >8 | 0.5 | 0.125 | 1 | 0.5 | 0.5 | >8 | 0.25 |
| **MTP113** | environnement | Montpellier, Herault, France | wood | PX211048 |  |  | 0.125 | 4 | 2 | >8 | >8 | 0.5 | 0.125 | 1 | 0.5 | 0.5 | >8 | 0.125 |

**Table S3**: Comparison of viability count (Colony Forming Unit/mL) obtained by three different inoculum preparation methods for seven *S. commune* strains: 1) covering the culture with 10 ml of sterile water as recommended by EUCAST (28); 2) manually vortexing one cm^2^ of fresh culture in 1.2 ml of sterile water in a haemolysis tube for 15 seconds at approximately 2,000 rpm; and 3) automatically fragmenting one cm^2^ of culture in a tube containing ceramic beads (MagNA Lyser Green Beads, Roche, REF 03358941001) and 1.2 ml of sterile water with an eccentric centrifuge (MagNA Lyser device) for 60 seconds at 2,000 rpm.

| n=7 | **Mean**  **(CFU/mL)** | **Standard deviation (CFU/mL)** | **Coefficient of variation (%)** |
| --- | --- | --- | --- |
| **Culture covering** | 6680 | 9660 | 145 |
| **Manual vortex** | 200 | 160 | 82 |
| **Automatic vortex  with bead-beating** | 1280 | 1320 | 103 |

**Table S4:** Repeatability test performed on 24 to 30 strains according to the antifungal agents tested. MICs were measured on duplicate on the same microdilution plate, the same day. Means, standard deviations and maximums are expressed in difference of dilution and not in µg/mL.

| *molecule* | **VOR** | **POS** | **ISA** | **ITR** | **TER** | **AMB** |
| --- | --- | --- | --- | --- | --- | --- |
| **Number of isolats** | 26 | 29 | 30 | 24 | 24 | 30 |
| **Mean** | 0.12 | 0.21 | 0.17 | 0.25 | 0 | 0.2 |
| **Standard deviation** | 0.33 | 0.41 | 0.46 | 0.44 | 0 | 0.41 |
| **Maximum** | 1 | 1 | 2 | 1 | 0 | 1 |

AMB, amphotericin B; ISA, isavuconazole; ITR, itraconazole; MIC, minimal inhibitory concentration; POS, posaconazole; TER, terbinafine; VOR, voriconazole.

**Table S5:** Reproducibility test **performed on 8 strains** of *Schizophyllum commune* according to the antifungal agents tested. MICs were measured on three different days from three different cultures plates of the same strains. The antifungal-strains couples with a difference of 2 dilutions are underlined in light grey. The antifungal-strain couple with a difference of 3 dilutions id highlighted in dark grey.

| *Molecule*  *Strain* | **VOR** | **POS** | **ISA** | **ITR** | **TER** | **AMB** |
| --- | --- | --- | --- | --- | --- | --- |
| **1** | 0.25-0.5-0.5 | 1-1-0.5 | 4-2-4 | 4-1-2 | >8->8->8 | 1-1-1 |
| **2** | 0.125-0.25-0.06 | 1-0.5-0.5 | 2-0.25-0.5 | 1-0.5-1 | >8->8->8 | 0.125-0.06-0.06 |
| **3** | 0.125-0.125-0.125 | 1-1-1 | 0.5-2-2 | 0.5-1-1 | >8->8->8 | 0.25-0.125-0.125 |
| **4** | 0.125-0.06-0.06 | 1-1-1 | 2-0.5-0.5 | 2-0.5-1 | >8->8->8 | 0.25-0.25-0.25 |
| **5** | 0.06-0.06-0.06 | 1-1-1 | 0.5-1-0.5 | 0.5-1-1 | >8->8->8 | 1-0.5-0.25 |
| **6** | 0.25-0.25-0.25 | 4-4-4 | 2-4-2 | 2-2-2 | >8->8->8 | 0.125-0.125-0.125 |
| **7** | 0.25-0.25-0.25 | 1-1-4 | 1-1-2 | 1-1-1 | >8->8->8 | 0.5-0.5-0.25 |
| **8** | 0.06-0.06-0.03 | 0.5-1-1 | 0.25-0.25-0.5 | 0.5-0.5-0.5 | >8->8->8 | 0.06-0.125-0.06 |

AMB, amphotericin B; ISA, isavuconazole; ITR, itraconazole; MIC, minimal inhibitory concentration; POS, posaconazole; TER, terbinafine; VOR, voriconazole.
